# Priority effects and competition by a native species inhibit an invasive species and may assist restoration

**DOI:** 10.1002/ece3.6938

**Published:** 2020-11-04

**Authors:** Hanxia Yu, Maofeng Yue, Cui Wang, Johannes J. Le Roux, Changlian Peng, Weihua Li

**Affiliations:** ^1^ Guangdong Provincial Key Laboratory of Biotechnology for Plant Development, Guangzhou Key Laboratory of Subtropical Biodiversity and Biomonitoring, School of Life Sciences South China Normal University Guangzhou China; ^2^ School of Biological and Food Engineering Guangdong University of Petrochemical Technology Maoming China; ^3^ Department of Biological Sciences Macquarie University Sydney NSW Australia

**Keywords:** biotic interactions, competitive exclusion, ecological restoration, *Mikania micrantha*, priority effect, *Pueraria lobata*

## Abstract

Selecting native species for restoration is often done without proper ecological background, particularly with regard to how native and invasive species interact. Here, we provide insights suggesting that such information may greatly enhance restoration success. The performance of the native vine, *Pueraria lobata*, and that of the invasive bitter vine, *Mikania micrantha*, were investigated in South China to test how priority effects (timing and rate of germination and seedling growth) and competition (phytochemical effects and competitive ability) impact invasive plant performance. We found that, in the absence of competition, the germination rate of *M. micrantha*, but not of *P. lobata*, was significantly affected by light availability. *P. lobata* seedlings also performed better than those of *M. micrantha* during early growth phases. Under competition, negative phytochemical effects of *P. lobata* on *M. micrantha* were strong and we found *M. micrantha* to have lower performance when grown with *P. lobata* compared to when grown by itself. Relative interaction indexes indicated that, under interspecific competition, *P. lobata* negatively affected (i.e., inhibited) *M. micrantha*, whereas *M. micrantha* positively affected (i.e., facilitated) *P. lobata*. Higher photosynthetic efficiency and soil nutrient utilization put *P. lobata* at a further advantage over *M. micrantha*. Field trails corroborated these experimental findings, showing little recruitment of *M. micrantha* in previously invaded and cleared field plots that were sown with *P. lobata*. Thus, *P. lobata* is a promising candidate for ecological restoration and for reducing impacts of *M. micrantha* in China. This research illustrates that careful species selection may improve restoration outcomes, a finding that may also apply to other invaded ecosystems and species.

## Graphical abstract

*P. lobata* negatively affected *M. micrantha*, whereas *M. micrantha* positively affected *P. lobata* under interspecific competition. *P. lobata* is a promising candidate for ecological restoration and for reducing impacts of *M. micrantha*.

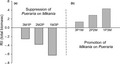

## 1 INTRODUCTION

Invasive plants pose significant threats to biodiversity, ecosystem services, regional economies, and public health (Castro‐Díez et al., 2016; Ricciardi et al., 2017; Simberloff, 2005). What makes some species invasive and others not remains an open question in ecology. This reflects the high context dependency of biological invasions. That is, species’ traits interact in complex ways with conditions in the new environment to determine the outcome of non‐native species introductions. These uncertainties also suggest that habitat‐specific approaches are needed to effectively manage invasive populations (Tang et al., 2013).

Competition‐based biotic resistance has been touted as an important mechanism to explain plant invasion success (Byun & Lee, 2017; MacDougall et al., 2009). Species‐rich communities will utilize resources more completely than species‐poor communities (Dimitrakopoulos & Schmid, 2004; Scherer‐Lorenzen et al., 2003), thereby limiting the resources available to new arrivals (Fargione et al., 2003; Hector et al., 2001). Under these circumstances, only highly competitive non‐native species may establish and become invasive. Species‐poor communities, for example, in recently disturbed habitats, may also repel invasive species if resident native species are strong competitors. Mwangi et al.'s (2007) found such resource “pre‐emption” to resist invasion during the early stages of succession. These authors showed that native species capable of earlier establishment, and that had faster growth rates, than non‐native species, pre‐empted resources, thereby negatively affecting slower‐growing non‐native species (Mwangi et al., 2007). These observations question the supposed link between high species richness and biotic resistance and suggest that resource pre‐emption, via priority effects and strong competition, may resist invasion, even in species‐poor communities.

From a restoration viewpoint, biotic resistance and resource pre‐emption also suggest that selecting native plants with low niche differentiation, but with higher competitive ability, from the most common invasive species, may enhance restoration success following the clearing of invasive species (Funk et al., 2008; Roberts et al., 2010; Young et al., 2005). However, native species used in restoration are often selected with limited ecological background, especially with regard to species interactions. Native species selection can also be difficult in habitats where no information is available on the original or “pristine” state of communities (Ostertag et al., 2015). In China, we observed the native leguminous vine, *Pueraria lobata* (Willd) Ohwi, and its cultivar *Pueraria lobata* var. *thomsonii* Benth Vaniot der Maesen (hereafter collectively referred to as *Pueraria*), to often co‐occur with the highly invasive vine, *Mikania micrantha* H. B. K. (hereafter referred to as *Mikania*). We also observed *Pueraria* seedlings to be generally taller than *Mikania* seedlings in the field, presumably because *Pueraria* seeds germinate earlier than *Mikania* seeds. *Pueraria* is widely distributed from South‐East Asia to Australia, and it has vigorous growth (up to 26 cm per day or 15 m per growing season), with new roots being produced wherever nodes make contact with soil (EPPO, 1997). *Pueraria* is also frequently used to improve soil because of its ability to fix nitrogen through rhizobium symbiosis (Li et al., 2015), making it a promising candidate for restoration.

Here, we tested how interactions between native *Pueraria* and invasive *Mikania* vines in China impact on the performance of each species. Specifically, we measured germination, seedling growth, phytochemical effects, and competitive interactions between these two vines. Our aim was to determine whether *Pueraria* can outperform *Mikania* through priority effects (via the timing of germination and seedling growth) and high competitiveness (via phytochemical effects and competitive ability), and therefore whether this it is a promising species for ecological restoration in areas previously invaded by *Mikania*.

## 2 MATERIALS AND METHODS

### 2.1 Seed germination and seedling growth performance

#### 2.1.1 Seed collection and scarification

Seeds of *Mikania* were collected during the flowering and seeding period (around November to December 2016) from a nursery site at Guangdong Academy of Agricultural Sciences, Guangzhou, China. Seeds of *P. lobata* var. *thomsonii* were bought from a commercial company. Half of the *Pueraria* seeds were soaked in 10 ml of 98% H_2_SO_4_ for 15–20 min, followed by rinsing with distilled water. The other half of *Pueraria* seeds and all *Mikania* seeds were sterilized by submersion in 10% sodium hypochlorite solution for 3 min followed by washing with distilled water.

#### 2.1.2 Response of seed germination to light

Photosensitivity of seed germination has a significant effect on the survival and establishment of plants (Finch‐Savage & Leubner‐Metzger, 2006). It was previously reported that *Mikania* seeds do not germinate easily under dark conditions and that the soil seed bank of this species produces very few seedlings in the absence of light (Li et al., 2002). Whether seed germination of *Pueraria* is dependent on the light is not clear. We designed an experiment to test to what extend light influences the germination of *Pueraria* and *Mikania* seeds.

Fifty sterilized *Mikania* seeds and 50 sterilized as well as 50 H_2_SO_4_‐treated seeds of *Pueraria* were put in a petri dish containing two layers of moist filter. Each seed type was replicated five times (*n* total = 30). Petri dishes were placed in incubators (RXZ Intelligent Type, Ningbo Jiangnan Instrument Factory, China) and subjected to one of two incubation conditions: (a) full illumination and darkness for 12 hr, at 30 and 27°C, day/night temperature, respectively; (b) darkness for 24 hr (30/27°C, day/night temperature). Humidity was maintained at 65% throughout the experiment. The number of germinated seeds was counted daily at 24 hr of intervals over a 14‐day period. We considered the emergence of radicles (>1 mm) from the seed coat as an indicator for successful germination. Finally, germination rate (GR) and germination index (GI) (Javaid & Tanveer, 2014) were calculated as follows:

GR=(Numberofgerminatedseeds/Totalnumberoftestedseeds)×100%

GI=Σ(Gt/Dt)(Gt‐Numberofgerminatedseedondayt,Dt‐dayt).

#### 2.1.3 Seedling growth

Fifty sterilized *Mikania* seeds and 50 sterilized as well as 50 H_2_SO_4_‐treated seeds of *Pueraria* were evenly sown in a pot (diameter: 12–19 cm, height: 15 cm) filled with nutrient‐rich soils. Each seed lot planting was replicated five times. Pots were covered with plastic film and kept in an artificial climate incubator (RXZ Intelligent, Ningbo Jiangnan Instrument Factory) set for full illumination and darkness for 12 hr each, and 30 and 27°C, day/night temperature, respectively, with 65% humidity. Once emerging seedlings broke the soil surface, the plastic film was removed and pots moved to the glasshouse located at the School of Life Sciences, South China Normal University. During the growth period, seedlings were thinned out to make sure that equal numbers of seedlings remained in all pots. Pots were watered every day and were randomized weekly. Plant height, number of leaves, and total biomass from five plants of each pot were measured every 15 days, and plants were harvested after 60 days of growth. Total biomass was calculated after drying plant material for at least 48 hr at 65°C in an oven.

#### 2.1.4 Phytochemistry

Allelopathy is an important mechanism of interspecific competition between plants, whereby phytochemicals released by one species inhibit the growth of another species (Rice, 1984). In order to compare the allelopathic potential of *Pueraria* and *Mikania*, a bioassay of aqueous phytochemical extracts on seed germination was carried out in a reciprocal experiment. Fresh stems and leaves of both species were collected from the biological garden in South China Normal University (23°8′N, 113°20′E). These were soaked in distilled water at room temperature for 48 hr. Extracts were diluted to obtain high (0.05 g/ml) and low (0.025 g/ml) phytochemical concentrations, while distilled water was used in control treatments. Fifteen milliliters of aqueous plant extract was added to a petri dish containing 50 seeds of the target species and kept in an incubator (RXZ Intelligent, Ningbo Jiangnan Instrument Factory) set at full illumination and darkness for 12 hr at 30 and 27°C day/night temperature, respectively, and a constant humidity of 65%. Each treatment was replicated five times. Moisture for each petri dish was maintained by adding equal amounts of treatment‐specific solution in subsequent days. Seedlings were photographed after 10 days to measure root length and seedling height using ImageJ software (ImageJ2x). Germination rate and germination indices were calculated as discussed above. The response index (RI) and synthetical effects (SE) provide more direct and accurate comparison for phytochemical effects between species (Williamson & Richardson, 1988). Response index was calculated as RI = (*T*/*C*) − 1, and synthetical effects (SE) as the average value of RI (Ma et al., 2006), where *T* is the treatment value, *C* is the control value. When *T* > *C*, RI > 0, it indicates the promoting effect; when *T* < *C*, RI < 0, it indicates the inhibiting effect, and the absolute value of RI represents the allelopathy intensity.

### 2.2 Plant performance and interspecific competition

#### 2.2.1 Experimental design

To assess the competitive interactions between *Mikania* and *Pueraria*, healthy seedlings of both species with identical growth and developmental stages were selected and transplanted in outdoor pots (18–28 cm in diameter, 21 cm in height). Surface soil (0–20 cm) in the biological garden of South China Normal University was collected and used as growth medium. The soil was broken up and mixed with appropriate amount of river sand and organic fertilizer to improve gas permeability and fertility. Planting arrangements included *Mikania*:*Pueraria* ratios of 4:0 (4M), 3:1 (3M1P), 2:2 (2M2P), 1:3 (1M3P), and 0:4 (4P); that is, each pot contained four seedlings. Each planting arrangement was replicated 15 times. All pots were put at a naturally lit experimental site in the biological garden, received equal amounts of nutrients and water, and were randomized every three days.

#### 2.2.2 Gas exchange and chlorophyll fluorescence

After 120 days of growth, three mature leaves of the same orientation (middle and upper leaves) from five plants of each treatment were selected and gas exchange and chlorophyll fluorescence measured. The net photosynthetic rate (*Pn*) and transpiration rate (*Tr*) were measured using a portable infrared gas analyzer Li‐6400 (LI‐COR, Inc.) at a photosynthetic photon flux density (PPFD) of 800 μmol m^−2^ s^−1^. At the same time, the maximum photochemical efficiency *Fv*/*Fm*, the effective quantum yield (Yield), and the electron transport rate (ETR) were measured at actual light intensity using a portable pulse‐modulated fluorometer (PAM‐2100, Walz, Effeltrich). Finally, water use efficiency (WUE = *Pn*/*Tr*) and Rubisco content (Rubisco [g/m^2^] = ETR × 0.014) were estimated (Evans & Poorter, 2001).

#### 2.2.3 Growth performance, competition, and soil nitrogen

Plant height, root length, number of branches, and biomass were determined for all five replicates at days 60, 90, and 120 following planting. Plant height and root length were measured with a measure tape (accuracy of 1 mm), and the number of branches was counted. Biomass was dried in oven until constant weight at 65°C before being weighed.

We calculated the relative interaction index (RII) (Armas et al., 2004; Domènech & Vilà, 2008; Li et al., 2015) to estimate the intensity of competition as RII = (*Bw* − *Bo*)/(*Bw* + *Bo*), where *Bw* is the individual biomass of the target plant when growing with another plant (i.e., under interspecific competition), and *Bo* is the individual biomass of the target plant when growing by itself (i.e., under intraspecific competition) after 120 days. RII has value range from −1 to +1, with negative values indicating competition (i.e., growth of the target species is reduced) while positive values indicate facilitation (i.e., growth of the target species is promoted).

Interspecific competition may change soil nutrient levels. Since *Pueraria* is a legume, it can elevate soil nitrogen availability through symbiosis with nitrogen‐fixing rhizobium. This may also result in changes in soil microbial biomass nitrogen. We compared soil nitrogen and the soil microbial biomass nitrogen between *Pueraria* and *Mikania*. For each species, plants from five pots (grown in monoculture) were excavated and rhizosphere soils collected after 120 days of growth. Total nitrogen content of these soils was determined using the Kjeldahl method (air‐dried soil passed through a 0.15‐mm sieve), while levels of ${\text{NH}}_{4}^{+}$ and ${\text{NO}}_{3}^{‐}$ were measured in fresh soil (passed through a 1‐mm sieve) by continuous flow analyzer (Proxima, Alliance Instruments). Soil microbial biomass nitrogen was measured using a chloroform fumigation method (Jenkinson & Powlson, 1976).

### 2.3 Field experiment

An experimental site was identified in a *Mikania* infested area in University Town, Guangzhou, China (lat. 23°3′N, long. 113°23′E). The region has a subtropical monsoonal climate with an annual mean temperature of 21–24°C and annual average precipitation of *ca*. 1,800 mm. The coverage of *Mikania* at the site was about 85%. Soil at the site contained 3.49 g/kg of soil organic matter (SOM), 0.28 g/kg of total nitrogen (TN), 1.91 mg/kg of NO_3_^‐^, 5.65 mg/kg of , 1.35 g/kg of total phosphorus (TP), 74.81 mg/kg of available phosphorus (AP), 5.51 mg/kg of microbial biomass nitrogen (MBN), and had a pH of 4.79.

Twenty‐four 2 m × 2 m plots, separated by at least 50 cm, were established at the study site. All above‐ground vegetation and most roots were removed in 12 plots to mimic *Mikania* control and removal. The remaining 12 plots were left untouched. Four sowing densities of *Pueraria* (0, 100, 200, and 400 grains/m^2^) were each replicated in three cleared and three uncleared plots. All seeds were treated with H_2_SO_4_ and sown after radicle emergence. *Pueraria* and *Mikania* plants in each plot were harvested six months after sowing and fresh above‐ground biomass (stem + leaf material) determined for each species. Samples were also oven‐dried to determine dry weight.

### 2.4 Statistical analysis

All statistical analyses were performed using SPSS 18.0 software (SPSS Inc.). Plant performance variables, gas exchange parameters, the fluorescence variables, and soil nitrogen indices between were compared between treatments using one‐way ANOVAs, followed by Duncan post hoc tests at *p* < .05. For germination rates and germination index analyses, significant differences under light and dark conditions were determined using *t* tests for two independent samples. We used linear regression to assess the response of total biomass and seedling height to planting time. Moreover, for total biomass and seedling height, significant differences between *Pueraria* and *Mikania* were determined using *t* tests for two independent samples in each stage throughout the growth period. General linear models were used to assess the effects of time and plant species as well as their interaction on total biomass and plant height during seedling growth period, and the effects of *Mikania* removal and sowing density of *Pueraria* seeds, as well as their interaction, on *Mikania* and *Pueraria* biomass under field conditions. The SigmaPlot 12.0 software was used to visualize data.

## 3 RESULTS

### 3.1 Response of seed germination to light seedling growth

The germination rate of *Pueraria* seeds was significantly lower than for *Mikania* seeds, especially under light conditions (Table 1). However, sulfuric acid treatment significantly improved both the germination rate and germination index of *Pueraria* seeds. *Pueraria* seeds (treated by H_2_SO_4_) germinated between 5.05 and 11.05 times faster than those of *Mikania*. The germination of *Pueraria* seeds was not affected by the light. Conversely, the germination rate and germination speed of *Mikania* were lower under the dark than under light conditions (Table 1).

**TABLE 1:** The comparison of germination of *Pueraria* and *Mikania* seeds (average ± *SD*, *n* = 5)

Species/Index	Germination rate (%)		Sig.	Germination index		Sig.
	Light	Dark		Light	Dark	
*Mikania* seeds	60.00 ± 0.07b	40.00 ± 0.12b	*	6.86 ± 0.74b	3.52 ± 1.17c	*
*Pueraria* seeds	32.67 ± 0.03c	32.67 ± 0.01b	ns	7.26 ± 0.76b	6.83 ± 0.91b	ns
*Pueraria* seeds treated by H_2_SO_4_	78.67 ± 0.01a	82.00 ± 0.02a	ns	34.63 ± 0.43a	38.89 ± 0.84a	ns

Note

Data with different letters for the same column indicate significant difference (one‐way ANOVA, followed by Duncan test, *p* < .05); *p < .05, ns‐no significance between light and dark, followed by *t* test of two independent samples.

**FIGURE 1:**
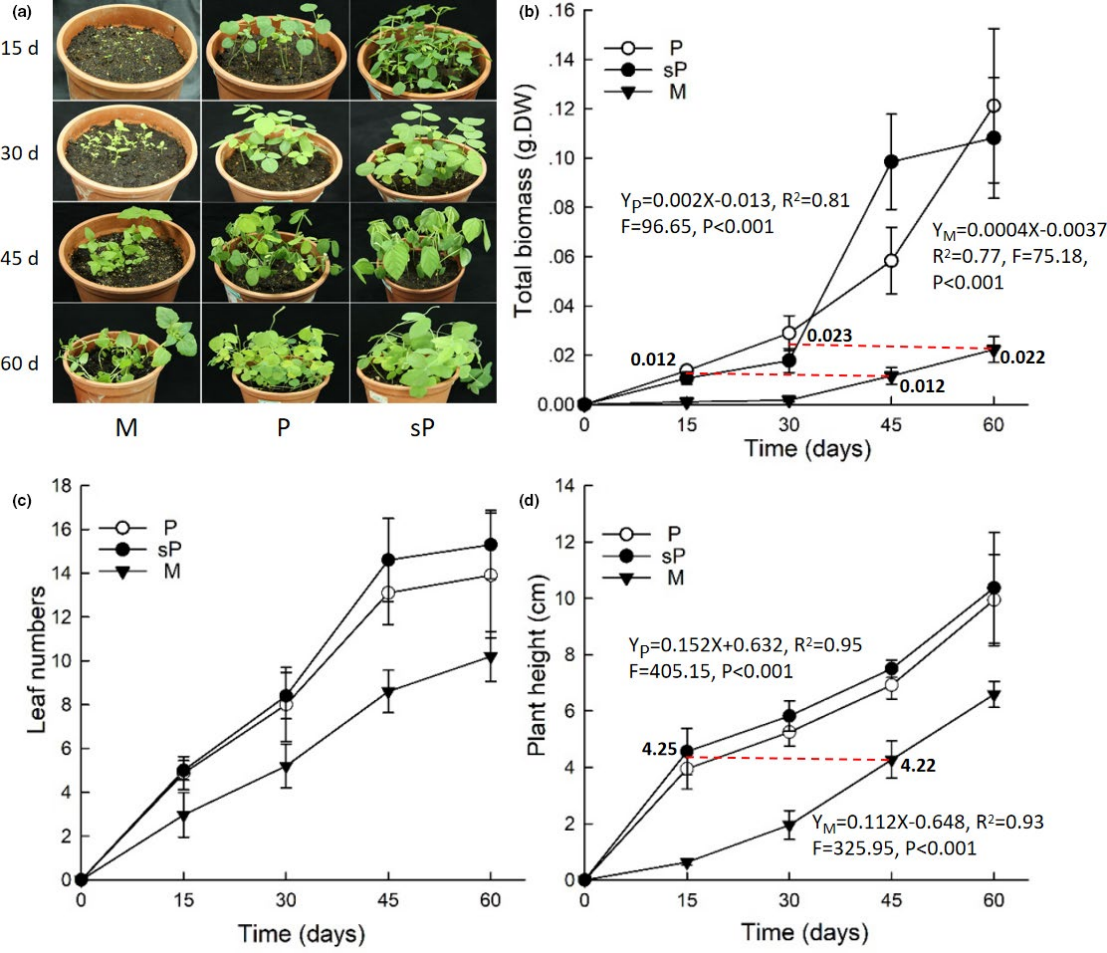
Changes in seedling growth of *Mikania* and *Pueraria* over time (average ± *SD*, *n* = 5). P – *Pueraria* seedlings; sP – *Pueraria* seedlings geminated from sulfuric acid‐treated seeds; M – *Mikania* seedlings. Red dotted lines indicate the equivalent level of the performance between *Mikania* and *Pueraria* seedling

Results from general linear models showed that growing time, plant species, and their interaction had a significant effect on total biomass and plant height. The effects of growing time and plant species on plant height were much greater than on total biomass, whereas their interaction on plant height was lower than on total biomass (Table 2). Both H_2_SO_4_‐treated and sterilized *Pueraria* seeds broke the soil surface about a week earlier than *Mikania* seeds. *Mikania* seedlings were small and slim at day 15, while *Pueraria* seedlings of the same age were taller and stronger. *Pueraria* seedlings from H_2_SO_4_‐treated seeds were the best in appearance at day 15 compared to all other seedlings (Figure 1a). The size of *Mikania* seedlings was only similar to 15‐day‐old *Pueraria* seedlings after 45 days of growth. *Pueraria* started to form tendrils after 60 days but not *Mikania* (Figure 1a). There were no significant differences in total biomass, number of leaves, and plant height between *Pueraria* seedlings, irrespective of scarification treatment, and both seed types performed better than *Mikania* (Figure 1b–d). Total biomass and plant height of 45‐day‐old *Mikania* seedlings were equivalent to that of *Pueraria* at 15 days (Figure 1b,d). Furthermore, total biomass of 60‐day‐old *Mikania* was equivalent to those of *Pueraria* at 30 days (Figure 1b). Furthermore, linear regression analysis and *t* test for two independent samples indicated that the growth rate of *Pueraria* seedlings was significantly higher than that of *Mikania* seedlings in each stage throughout the growth period.

**TABLE 2:** Results of general linear models assessing the effects of time and plant species as well as their interaction on total biomass and plant height during seedling growth period

Independent variables	Total biomass			Plant height		
	*df*	*F*	*p*	*df*	*F*	*p*
Time	4	68.98	.000	4	430.37	.000
Plant species	1	142.03	.000	1	336.48	.000
Time * Plant species	4	31.22	.000	4	21.66	.000

### 3.2 Phytochemistry

Aqueous phytochemical extracts of *Pueraria* inhibited the germination and seedling growth of *Mikania*, irrespective of concentration (Table 3). This potential allelopathic effect was stronger for extracts from *Pueraria* stems than extracts from leaves. The overall synthetical effect value of *Pueraria* on *Mikania* was −1.39.

**TABLE 3:** The synthetical effects of the aqueous extract of *Pueraria* on seed germination and seedling growth of *Mikania* (average ± *SD*, *n* = 5)

*Pueraria*	Leaves		Stems		Overall
Phytochemicals	0.025 g/ml	0.05 g/ml	0.025 g/ml	0.05 g/ml	
RI (germination rate)	−0.05	−0.03	−0.09	−0.22	
RI (germination index)	−0.07	−0.04	−0.06	−0.13	
RI (root length)	−0.34	−0.27	−0.43	−0.68	
RI (height)	0.20	0.22	0.24	0.36	
Synthetical effects	−0.26	−0.12	−0.34	−0.67	−1.39

Aqueous extracts of *Mikania* leaves and stems with lower concentration had inhibitory effects on the seed germination and seedling growth of *Pueraria;* however, its stem aqueous extract with high concentration promoted seed germination of *Pueraria* (Table 4). The overall synthetical effect value of *Mikania* on *Pueraria* was −0.64. The allelopathic inhibition of *Pueraria* on *Mikania* is therefore stronger than that of *Mikania* on *Pueraria*.

**TABLE 4:** The synthetical effects of the aqueous extracts of *Mikania* on seed germination and seedling growth of *Pueraria* (average ± *SD*, *n* = 5)

*Mikania*	Leaves		Stems		Overall
Phytochemicals	0.025 g/ml	0.05 g/ml	0.025 g/ml	0.05 g/ml	
RI (germination rate)	0.02	0.00	−0.06	0.11	
RI (germination index)	−0.16	−0.08	−0.12	0.12	
RI (root length)	−0.17	−0.29	−0.13	−0.17	
RI (height)	0.09	0.02	0.12	0.06	
Synthetical effects	−0.22	−0.35	−0.19	0.12	−0.64

### 3.3 Plant performance and interspecific competition

**FIGURE 2:**
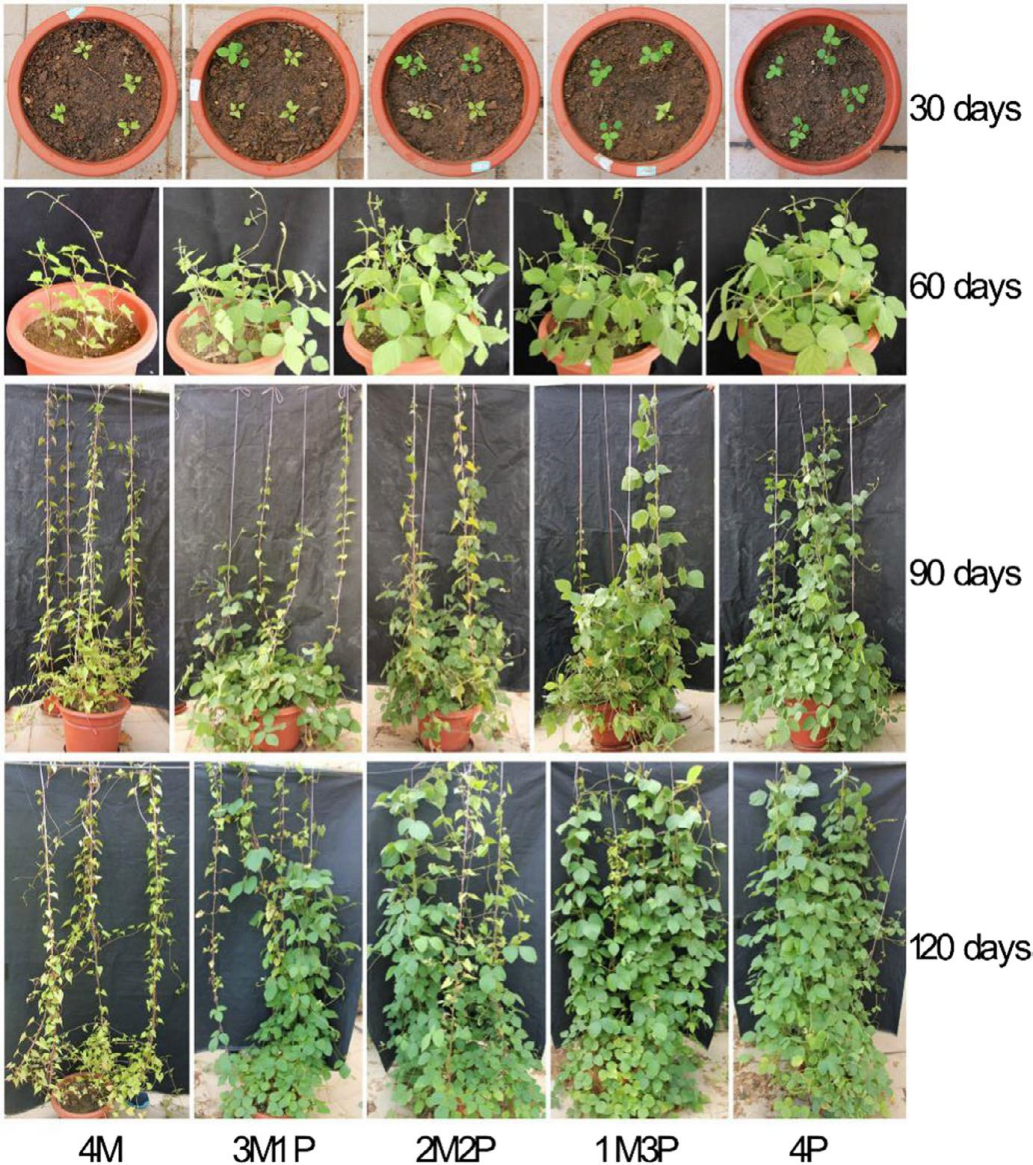
Changes in phenotypic characteristics under interspecific competition (average ± *SD*, *n* = 15). 4M – four *Mikania* seedlings planted by itself; 3M1P – three *Mikania* seedlings planted with one *Pueraria* seedling; 2M2P – two *Mikania* seedlings planted with two *Pueraria* seedlings; 1M3P – one *Mikania* seedling planted with three *Pueraria* seedlings; 4P – four *Pueraria* seedlings planted by itself

*Mikania* had slow growth during the first month. For the next 2–4 months, *Pueraria* showed much higher performance than *Mikania* under interspecific competition, irrespective of planting arrangement (Figure 2).

#### 3.3.1 *Mikania* growth performance

In early and mid‐growth stages (60 days and 90 days, respectively), there was no significant difference in root length of *Mikania* plants between treatments (Table 5). In later growth stages (i.e., 120 days), root length in higher mixed ratios of *Pueraria* to *Mikania*, that is, 1M3P and 2M2P, was significantly lower than for the 3M1P ratio or when grown by itself (4M) (Table 5). This was similar for plant height between 60 and 90 days of growth, being significantly lower in all the three competition treatments, that is, 3M1P, 2M2P, and 1M3P, compared to when grown by itself (4M) (Table 5). In the competition experiment, as the ratio of *Pueraria* to *Mikania* increased, *Mikania* plant height decreased significantly following 120 days of growth (Table 5). *Mikania* did not branch after the first 60 days of growth, and there were no significant differences in the number of branches between treatments until 90 days of growth. Branch numbers of *Mikania* also declined gradually as the ratio of *Pueraria* to *Mikania* increased at day 120 (Table 5).

**TABLE 5:** Changes of *Mikania* growth index under interspecific competition (average ± *SD*, *n* = 5)

Growth index	Time (days)	4M	3M1P	2M2P	1M3P
Root length (cm)	60	27.05 ± 3.92a	25.11 ± 2.01a	28.23 ± 2.57a	25.90 ± 5.14a
	90	43.73 ± 4.02a	45.38 ± 9.26a	47.30 ± 9.90a	52.53 ± 7.13a
	120	48.75 ± 9.43a	39.76 ± 6.05b	30.79 ± 3.72c	26.32 ± 2.10c
Height (cm)	60	30.64 ± 6.49a	18.24 ± 2.20b	20.72 ± 2.40b	17.26 ± 3.40b
	90	161.53 ± 27.53a	114.82 ± 17.43b	109.00 ± 14.01b	83.53 ± 15.65c
	120	223.25 ± 25.52a	199.92 ± 30.23b	173.17 ± 38.60c	102.10 ± 16.64d
Branches	60	0	0	0	0
	90	4.3 ± 1.15a	3.3 ± 1.12a	3.0 ± 0.89a	2.7 ± 1.15a
	120	8.4 ± 1.60a	7.3 ± 1.44ab	6.6 ± 1.51b	4.2 ± 1.30c

Note

4M – four *Mikania* seedlings planted by itself; 3M1P – three *Mikania* seedlings planted with one *Pueraria* seedling; 2M2P – two *Mikania* seedlings planted with two *Pueraria* seedlings; 1M3P – one *Mikania* seedling planted with three *Pueraria* seedlings. Means within the same row followed by different small letters indicate significant difference at *p* < .05 (Duncan test).

**FIGURE 3:**
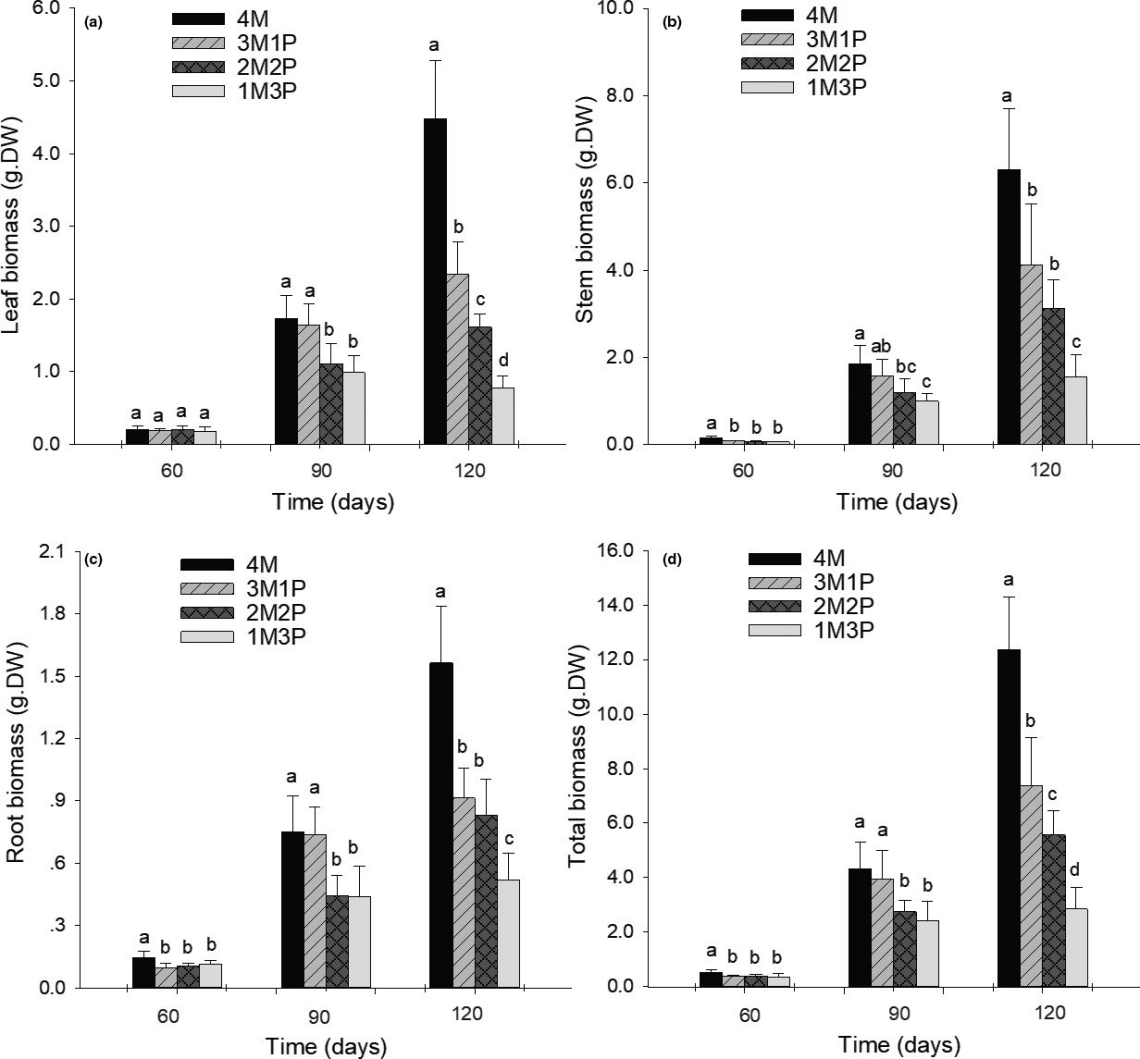
Changes in *Mikania* biomass under interspecific competition (average ± *SD*, *n* = 5). 4M – four *Mikania* seedlings planted by itself; 3M1P – three *Mikania* seedlings planted with one *Pueraria* seedling; 2M2P – two *Mikania* seedlings planted with two *Pueraria* seedlings; 1M3P – one *Mikania* seedling planted with three *Pueraria* seedlings; 4P – four *Pueraria* seedlings planted by itself. Different letters above the error bars indicate significant difference at *p* < .05 (Duncan test)

Total biomass, stem biomass, and root biomass of *Mikania* were significantly lower in all three competition treatments compared to when grown alone after 60 days of growth (Figure 3). These biomass fractions were significantly lower in the 2M2P and 1M3P than in the 4M and 3M1P planting arrangements after 90 days of growth (Figure 3). Stem biomass decreased gradually from 4M to 1M3P over the same period. Changes in the total biomass and leaf biomass significantly decreased in the order of: 1M3P < 2M2P < 3M1P < 4M, following 120 days of growth (Figure 3). Stem biomass and root biomass showed a similar trend (1M3P < 2M2P ~ 3M1P < 4M) as the mix ratio of *Pueraria* to *Mikania* increased (Figure 3).

#### 3.3.2 *Pueraria* growth performance

In early growth stages (i.e., after 60 days), there was no significant difference in root length of *Pueraria* seedlings between planting arrangements (Table 6). In mid‐growth stages (i.e., after 90 days), seedling root length in 1P3M was significantly higher than for the other treatments and in late growth stages (i.e., after 120 days of growth), and *Pueraria* root length gradually increased with an increase in *Mikania* to *Pueraria* planting ratios (Table 6). Variation in plant height followed a similar pattern (Table 6). Plant height of *Pueraria* in all the three competition treatments was higher than that of *Pueraria* grown by itself (4P) after 90 days of growth, and increased significantly as the ratios of *Mikania* to *Pueraria* increased after 120 days of growth (Table 6). The number of branches of *Pueraria* did not differ for the first 90 days and at 120 days between treatments, but they were higher in higher ratios of *Mikania* to *Pueraria* (1P3M and 2P2M) than in lower ratios (3P1M and 4P; Table 6).

**TABLE 6:** Changes in *Pueraria* growth index under interspecific competition (average ± *SD*, *n* = 5)

Growth index	Time (days)	4P	3P1M	2P2M	1P3M
Root length (cm)	60	30.93 ± 6.79a	32.58 ± 5.10a	38.10 ± 7.86a	41.97 ± 10.82a
	90	53.23 ± 9.81b	47.77 ± 9.48b	56.72 ± 13.45b	71.00 ± 5.07a
	120	54.54 ± 5.08b	58.23 ± 8.02ab	62.66 ± 10.24ab	65.14 ± 8.57a
Height (cm)	60	36.46 ± 6.03a	41.07 ± 7.57a	32.50 ± 7.83a	38.10 ± 1.92a
	90	110.26 ± 19.40b	138.11 ± 13.08a	138.40 ± 18.49a	149.93 ± 11.68a
	120	195.00 ± 20.12c	219.33 ± 19.20b	244.08 ± 24.97a	245.82 ± 41.79a
Branches	60	2.8 ± 0.39a	2.8 ± 0.44a	2.7 ± 0.52a	2.7 ± 0.58a
	90	7.6 ± 1.98a	7.3 ± 1.80a	8.0 ± 1.26a	7.7 ± 1.15a
	120	9.3 ± 1.74b	8.6 ± 1.59b	13.1 ± 2.64a	12.8 ± 2.05a

Note

4P – four *Pueraria* planted by itself; 3P1M – three *Pueraria* seedlings planted with one *Mikania* seedling; 2P2M – two *Pueraria* seedlings planted with two *Mikania* seedlings; 1P3M – one *Pueraria* seedling planted with three *Mikania* seedlings; means within the same row followed by different small letters indicate significant difference at *p* < .05 (Duncan test).

**FIGURE 4:**
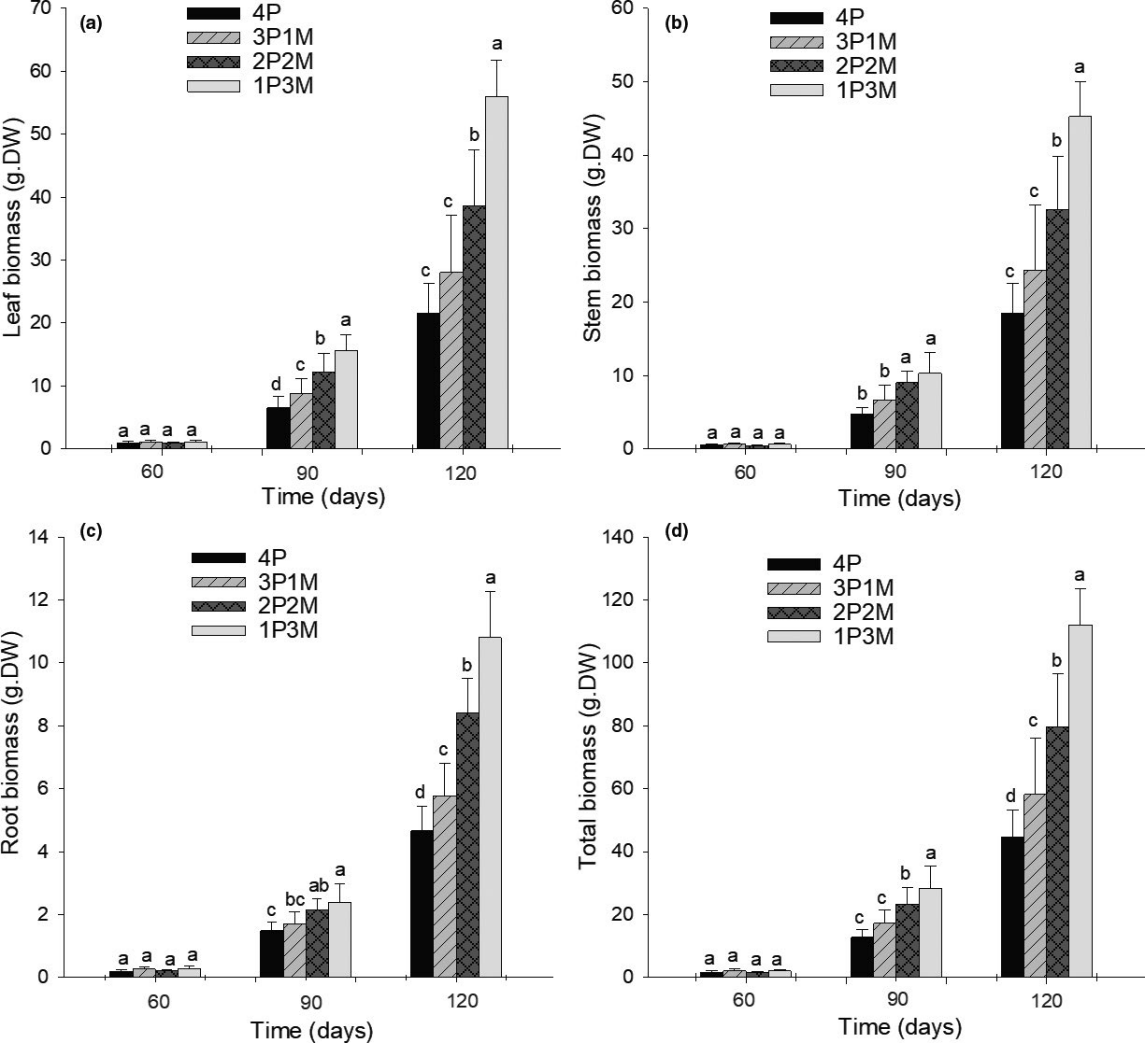
Changes in *Pueraria* biomass under interspecific competition (average ± *SD*, *n* = 5). 4M – four *Mikania* seedlings planted by itself; 3M1P – three *Mikania* seedlings planted with one *Pueraria* seedling; 2M2P – two *Mikania* seedlings planted with two *Pueraria* seedlings; 1M3P – one *Mikania* seedling planted with three *Pueraria* seedlings; 4P – four *Pueraria* seedlings planted by itself. Different letters above the error bars indicate significant difference at *p* < .05 (Duncan test)

We found no significant differences in the biomass production of *Pueraria* between treatments during early growth stages (i.e., after 60 days; Figure 4). In mid‐growth stages (i.e., 90 days), *Pueraria* biomass started to increase gradually as the ratio of *Mikania* to *Pueraria* increased (Figure 4). After 120 days of growth, *Pueraria* grown with high‐density *Mikania* (i.e., 1P3M) produced the most biomass (Figure 4).

#### 3.3.3 Relative interaction index (RII)

**FIGURE 5:**
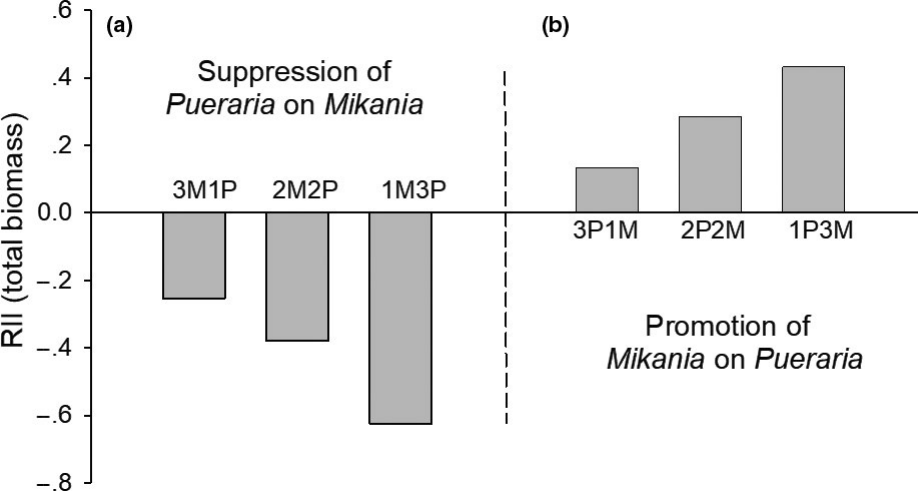
Comparison of relative interaction index (RII) between *Pueraria* and *Mikania* grown for 120 days. 3M1P – three *Mikania* seedlings planted with one *Pueraria* seedling; 2M2P – two *Mikania* seedlings planted with two *Pueraria* seedlings; 1M3P – one *Mikania* seedling planted with three *Pueraria* seedlings; 3P1M – three *Pueraria* seedlings planted with one *Mikania* seedling; 2P2M – two *Pueraria* seedlings planted with two *Mikania* seedlings; 1P3M – one *Pueraria* seedling planted with three *Mikania* seedlings

The relative interaction index of *Mikania*, when grown in the presence of *Pueraria*, was negative (RII_3M1P_ = −0.253, RII_2M2P_ = −0.379, RII_1M3P_ = −0.625), indicating an inhibitory effect by *Pueraria* on *Mikania* growth (Figure 5a). Furthermore, the magnitude of these negative effects increased with increased ratios of *Pueraria* to *Mikania*. *Mikania* showed an opposite trend, positively effecting the growth of *Pueraria* (RII_3P1M_ = 0.131, RII_2P2M_ = 0.281, RII_1P3M_ = 0.430). The magnitude of these positive effects increased as *Mikania* to *Pueraria* ratios increased in planting arrangements (Figure 5b).

#### 3.3.4 Gas exchange and chlorophyll fluorescence

**FIGURE 6:**
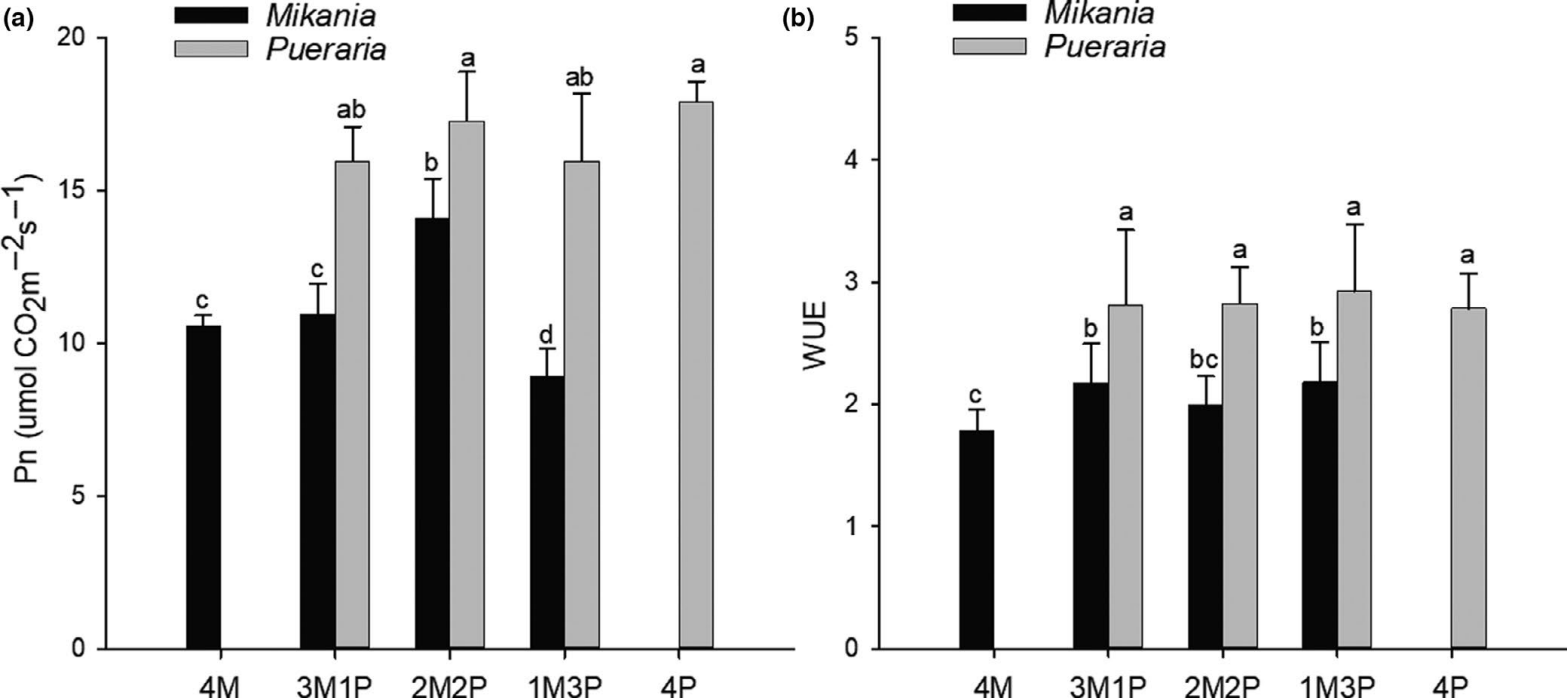
Changes in net photosynthetic rate (*Pn*) and water use efficiency (WUE) under interspecific competition (average ± *SD*, *n* = 9). 4M – four *Mikania* seedlings planted by itself; 3M1P – three *Mikania* seedlings planted with one *Pueraria* seedling; 2M2P – two *Mikania* seedlings planted with two *Pueraria* seedlings; 1M3P – one *Mikania* seedling planted with three *Pueraria* seedlings; 4P – four *Pueraria* seedlings planted by itself. Different letters above the error bars indicate significant difference at *p* < .05 (Duncan test)

The net photosynthetic rate (Pn) of *Pueraria* was significantly higher than that of *Mikania* when grown alone or under interspecific competition (Figure 6a). Competition did not affect the Pn of *Pueraria* but reduced the Pn of *Mikania* when the mix ratio of *Pueraria* to *Mikania* reached 3:1 (Figure 6a). Water use efficiency (WUE) showed a similar response with the WUE of *Pueraria* being significantly higher than that of *Mikania* under various planting arrangements (Figure 6b).

When *Mikania* was grown alone or in lower mix ratios of *Pueraria* to *Mikania* (1:3), the maximal photochemical efficiency (*Fv*/*Fm*) of dark‐relaxed Photosystem II (the photosystem that splits water to evolved oxygen) decreased significantly compared to that of *Mikania* grown with higher mix ratios of *Pueraria* to *Mikania* (2:2 and 3:1) (Table 7). However, the photochemical yield (Yield) of photosystem II of *Mikania* under illumination and the highest *Pueraria* to *Mikania* mix ratio (i.e., 3:1) was significantly lower than in other planting arrangements (Table 7). The electron transport rate (ETR) of *Mikania*, estimated by chlorophyll fluorescence, decreased significantly as the mix ratio of *Pueraria* to *Mikania* increased. Changes in Rubisco (the enzyme complex that fixes CO_2_ in plant leaves) showed a similar trend (Table 7). However, *Pueraria* showed the opposite trend for chlorophyll fluorescence parameters. First, Fv/Fm of *Pueraria* was not affected by any of the competition treatments. Secondly, Yield, ETR, and Rubisco of *Pueraria* increased significantly as *Mikania* to *Pueraria* mix ratios increased (Table 7).

**TABLE 7:** Changes in chlorophyll fluorescence parameters under interspecific competition (average ± *SD*, *n* = 5)

Plant species	*Fv*/*Fm*	Yield	ETR (µmol m^−2^ s^−1^)	Rubisco (g/m^2^)
1M 3P	0.807 ± 0.01a	0.14 ± 0.03b	44.96 ± 3.34d	0.63 ± 0.05d
2M 2P	0.803 ± 0.01a	0.23 ± 0.02a	52.80 ± 2.37c	0.74 ± 0.03c
3M 1P	0.784 ± 0.03b	0.26 ± 0.01a	66.24 ± 1.24b	0.93 ± 0.02b
4M	0.799 ± 0.01b	0.24 ± 0.01a	86.42 ± 3.42a	1.21 ± 0.05a
1P 3M	0.825 ± 0.02a	0.43 ± 0.03a	118.42 ± 0.79a	1.66 ± 0.01a
2P 2M	0.831 ± 0.01a	0.37 ± 0.02b	71.84 ± 1.63b	1.01 ± 0.02b
3P 1M	0.828 ± 0.01a	0.23 ± 0.04c	60.90 ± 1.24c	0.85 ± 0.02c
4P	0.826 ± 0.01a	0.19 ± 0.01d	45.80 ± 2.74d	0.64 ± 0.04d

Note

1M3P – one *Mikania* seedling planted with three *Pueraria* seedlings; 2M2P – two *Mikania* seedlings planted with two *Pueraria* seedlings; 3M1P – three *Mikania* seedlings planted with one *Pueraria* seedling; 4M – four *Mikania* seedlings planted by itself; 1P3M – one *Pueraria* seedling planted with three *Mikania* seedlings; 2P2M – two *Pueraria* seedlings planted with two *Mikania* seedlings; 3P1M – three *Pueraria* seedlings planted with one *Mikania* seedlings; 4P – four *Pueraria* planted by itself. Means within the same column (divided two groups, upper and lower parts as one independent group, respectively) followed by different small letters indicate significant difference at *p* < .05 (Duncan test).

#### 3.3.5 Soil nitrogen characteristics

Following 120 days of growth, ammonium and microbial biomass nitrogen (MBN) were the highest in *Pueraria* soils among the three soils we analyzed, while nitrate nitrogen and total nitrogen were higher in *Pueraria* soils than in *Mikania* soils. There were no significant differences in nitrate nitrogen and total nitrogen between *Pueraria* soils and the initial soils (Table 8).

**TABLE 8:** Soil nitrogen characteristics after *Mikania* and *Pueraria,* respectively, planted alone for 120 days in the pot experiment (average ± *SD*, *n* = 3)

Soil samples	NH_4_^+^‐N (mg/kg)	MBN (mg/kg)	NO_3_^‐^‐N (mg/kg)	TN (g/kg)
Initial soil	5.15 ± 0.81b	53.60 ± 13.70c	8.58 ± 1.09a	0.93 ± 0.05a
*Mikania* soil	4.75 ± 1.41b	95.60 ± 4.90b	7.03 ± 1.06b	0.81 ± 0.08b
*Pueraria* soil	27.15 ± 1.12a	141.50 ± 16.6a	8.90 ± 1.35a	0.97 ± 0.06a

Note

Data with different letters in the same column indicate significant difference at *p* < .05 (Duncan test).

Abbreviations: MBN, microbial biomass nitrogen; TN, total nitrogen.

### 3.4 Field experiment

**FIGURE 7:**
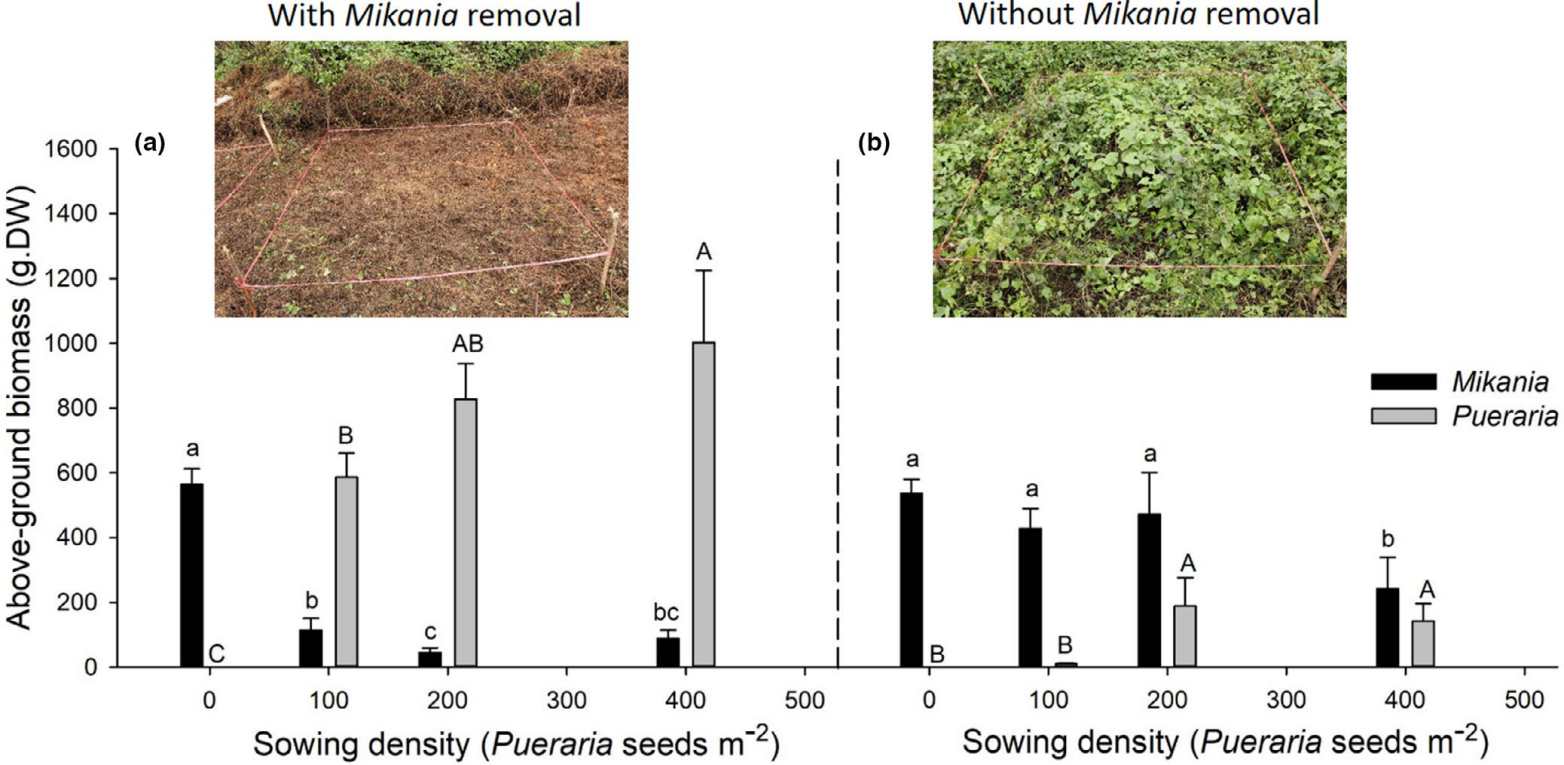
*Mikania* regeneration and *Pueraria* performance after sowing *Pueraria* seeds in the field plots (average ± *SD*, *n* = 3). Lowercase letters above the black columns indicate significant difference in *Mikania* biomass, and capital letters above the gray columns indicate significant difference in *Pueraria* biomass at *p* < .05 (Duncan test)

Following clearing and sowing of *Pueraria* seeds in the field, we found that the regeneration of *Mikania* declined dramatically after six months (by −79.8%, −92.0% and −84.3%, at sowing densities of 100, 200, and 400 *Pueraria* seeds/m^2^, respectively) compared to the control sites. *Pueraria* biomass increased significantly with increased sowing density (Figure 7a). Similar trends were observed when *Pueraria* seeds were sown in soils without the removal of *Mikania*. Although not as dramatic, the regeneration of *Mikania* significantly decreased by up to 55.0% when *Pueraria* was sown at a density of 400 seeds/m^2^ (Figure 7b). *Mikania* regeneration was lowest in plots where *Mikania* was removed and when *Pueraria* sowing density was 200 seeds/m^2^, while regeneration decreased significantly under a sowing density of 400 seeds/m^2^ in plots without *Mikania* removal.

Results from general linear models showed that removal, sowing density, and their interaction had a significant effect on above‐ground biomass of both *Mikania* and *Pueraria*. The effect of *Mikania* removal treatments was higher than that of sowing density on biomass of both vine species, whereas the removal of *Mikania* had a lower impact on *Mikania* than *Pueraria* performance (Table 9).

**TABLE 9:** Results of general linear models assessing the effects of *Mikania* removal and sowing density as well as their interaction on *Mikania* biomass and *Pueraria* biomass in the field

Independent variables	*Mikania* biomass			*Pueraria* biomass		
	*df*	*F*	*p*	*df*	*F*	*p*
*Mikania* removal	1	60.70	.000	1	166.50	.000
Sowing density	3	36.05	.000	3	41.10	.000
*Mikania* removal * sowing density	3	12.77	.000	3	20.78	.000

## 4 DISCUSSION

Understanding the ecological mechanisms that underlie the invasion success of non‐native species has attracted much research attention, yet surprisingly little is known on how these processes may inform management and the restoration of invaded habitats. Using native and invasive vines, we provide evidence to suggest that with careful selection of native species, based on intrinsic ecological and physiological attributes, enhanced ecological restoration outcomes may be achieved.

### 4.1 Differences between native and invasive species as modulated by abiotic conditions

Abiotic environmental conditions represent a significant barrier to the establishment success of, and subsequent invasion by, non‐native species (Blackburn et al., 2011). We found the rate and speed of germination of scarified *Pueraria* seeds to be much higher than for *Mikania* seeds, independent of light exposure. *Pueraria* seedlings were also taller and stronger than *Mikania* during early growth, becoming more evident over time, as indicated by total biomass accumulation, number of leaves produced, and plant height. *Pueraria* seeds germinated one week earlier than *Mikania* seeds and the growth performance of *Mikania* seedlings lagged behind *Pueraria* seedlings for at least 1 month. Thus, abiotic conditions differentially affected the performance of these two vines and early emergence, fast germination, and seedling growth may lead to a priority effect of *Pueraria* on *Mikania*, and possibly to the pre‐emption of resources. Early emergence is also expected to increase other components of plant fitness such as seedling growth (Verdu & Traveset, 2005). Others have previously found native species to similarly suppress the performance of invaders, suggesting that the ability of pioneer native species to rapidly colonize available habitat may suppress invasive species (Firn et al., 2010).

### 4.2 Interspecific competition between *Mikania* and *Pueraria*

Both our phytochemical and interspecific competition experiments supported the hypothesis of competitive exclusion of invasive *Mikania* by native *Pueraria*. Plants can affect their neighbors in numerous ways through the release of phytochemical compounds into the environment (He et al., 2012). For example, inhibitory allelopathic chemicals are widely reported as major contributors to enhanced interspecific competition (Bais et al., 2003; Callaway & Ridenour, 2004; Inderjit et al., 2008; Yuan et al., 2013). Our results showed that the total synthetical effects of *Pueraria* on *Mikania* were much higher than vice versa, indicating strong inhibitory effects of *Pueraria* on *Mikania*. A possible explanation for this could be the release of allelochemicals by *Pueraria* which enhance its competitive ability (i.e., interference competition) over *Mikania* by affecting the early growth performance of *Mikania*. Others have also found *Mikania* to be very sensitive to allelopathic chemicals produced by native plants (e.g., *Dicranopteris pedate;* Zhao et al., 2008).

The higher performance of *Pueraria* compared to *Mikania* that we observed for germination and seedling growth, as well as allelopathy, was also mirrored in plant performance under interspecific competition. That is, *Pueraria* negatively affected *Mikania* while *Mikania* had the opposite effect on *Pueraria*. Previous work also found sweet potato to have similar effects on *Mikania* (Shen et al., 2015). Both *Pueraria* and *Mikania* are perennial evergreen vines with large capacities for asexual propagation, and the two species share many morphological similarities, occupying virtually the same niche in agroforestry systems (Li et al., 2012; Lian et al., 2006). Our results provide some evidence for competitive exclusion of *Mikania* by *Pueraria*, suggesting that native species with similar traits, and with similar niche requirements to non‐native species, may impede invasion (Emery, 2007; MacDougall et al., 2009). Tillering, branching, and plant height are important traits that facilitate competition in plants, and have been considered important for pre‐empting resources during scramble competition (Jiang et al., 2008). The branch number and plant height of *Mikania* in the present study markedly decreased with higher planting ratios of *Pueraria* to *Mikania*. It is noteworthy that *Pueraria* is also a highly successful invasive species outside of its native range (Bentley & Mauricio, 2016; Kartzinel et al., 2015).

The apparent superior competitive ability of *Pueraria* could also be related to its photosynthetic characteristics. We found that gas exchange and chlorophyll fluorescence parameters give *Pueraria* an apparent advantage over *Mikania*. *Pueraria* produced much more leaf biomass per plant than *Mikania* under interspecific competition, which resulted in partial shading of the latter's leaves. The lower growth irradiance to which *Mikania* leaves were exposed due to lower light availability under these conditions may also cause lower Rubisco and cytochrome *bf* content. The latter is often a rate‐limiting bottleneck in electron flow from PSII to PSI (Anderson et al., 1988). Since *Mikania* is intrinsically heliophilic, with a high light compensation point of photosynthesis (1,002 μmol m^−2^ s^−1^) (Wen et al., 2000), it is capable of rapid growth under high light conditions. However, there is a sharp decline in this response under shaded conditions (Liao et al., 2003), making it be vulnerable to competition.

Our recent work also found *Mikania* invasion to lead to substantial losses of soil nitrogen (Liu et al., 2020), which provides a good opportunity for soil remediation by leguminous plants such as *Pueraria*. We found ammonium and microbial biomass nitrogen in *Pueraria* soils to be high at later growth stages (120 days). However, nitrate and total nitrogen did not change over the same time period in these soils. On the other hand, nitrate and total nitrogen were higher in *Pueraria* than *Mikania* soils. This may reflect the ability of *Pueraria* to fix atmospheric nitrogen through its association with rhizobium bacteria. This may provide *Pueraria* with a further competitive advantage over *Mikania* when competing for available nitrogen sources essential for rapid growth.

Our field data corroborated our potting experiments. We found that the removal of *Mikania* more severely impacted its performance than *Pueraria* sowing density, so management interventions require both the removal of *Mikania* biomass and active restoration. Similar competitive effects of *Pueraria* on other invasive vines have been observed, for example, we previously found the recruitment of invasive *Ipomoea cairica* to be severely hampered by *Pueraria* (Li et al., 2015).

## 5 CONCLUSIONS

Through competitive exclusion, native species with superior fitness may limit the establishment and spread of non‐native species. However, competitive exclusion is not widely applied to control invasive plants (Byun & Lee, 2017). We found that plant traits such as seed germination, seedling growth speed, biomass accumulation, branching, plant height, photosynthetic efficiency, and the direction and magnitude of phytochemical effects, all played a significant role in the competitive exclusion of *Mikania* by *Pueraria*. Evidently, *Pueraria* can outperform *Mikania*, modulated by priority effects and strong competition. Although *Pueraria* is regarded as an invasive species elsewhere (Bentley & Mauricio, 2016; Kartzinel et al., 2015), it is a valuable native plant in China. The starch‐rich roots of *Pueraria* are valued as a food source and for medicinal purposes. Thus, planting *Pueraria* is a promising approach to reduce the extent of *Mikania* invasion in tropical and subtropical agroforestry systems that are suitable for *Pueraria* cultivation, simultaneously providing weed management and economic benefits. Overall, this study provided promising strategies for species selection for restoration. Specifically, restoration will benefit from (a) the selection of highly competitive native species that have high niche overlap with invasive species, (b) the selection of native species that show strong priority effects (e.g., early emergence, rapid germination, and seedling growth), (c) selecting economically valuable native legumes that can improve restored soils through nitrogen fixation. Our study also provides a valuable framework that may be applied to other invaded ecosystems and species.

## CONFLICT OF INTEREST

None declared.

## AUTHOR CONTRIBUTION

**Hanxia Yu:** Data curation (equal); Writing‐review & editing (equal). **Maofeng Yue:** Data curation (equal); Funding acquisition (equal). **Cui Wang:** Investigation (equal). **Johannes Le Roux:** Writing‐review & editing (equal). **Chang Lian Peng:** Writing‐review & editing (equal). **Weihua Li:** Conceptualization (equal); Methodology (equal); Supervision (equal); Writing‐original draft (equal); Writing‐review & editing (equal).

## Data Availability

All the data used in this manuscript are available from https://osf.io/vq9kw/.
